# *De novo* Transcriptome Assemblies of *Rana* (*Lithobates*) *catesbeiana* and *Xenopus laevis* Tadpole Livers for Comparative Genomics without Reference Genomes

**DOI:** 10.1371/journal.pone.0130720

**Published:** 2015-06-29

**Authors:** Inanc Birol, Bahar Behsaz, S. Austin Hammond, Erdi Kucuk, Nik Veldhoen, Caren C. Helbing

**Affiliations:** 1 Canada’s Michael Smith Genome Sciences Centre, British Columbia Cancer Agency, Vancouver, BC, V5Z 4S6, Canada; 2 Department of Biochemistry and Microbiology, University of Victoria, P.O. Box 1700, Stn CSC, Victoria, BC, V8W 2Y2, Canada; University Claude Bernard Lyon 1, FRANCE

## Abstract

In this work we studied the liver transcriptomes of two frog species, the American bullfrog (*Rana* (*Lithobates*) *catesbeiana*) and the African clawed frog (*Xenopus laevis*). We used high throughput RNA sequencing (RNA-seq) data to assemble and annotate these transcriptomes, and compared how their baseline expression profiles change when tadpoles of the two species are exposed to thyroid hormone. We generated more than 1.5 billion RNA-seq reads in total for the two species under two conditions as treatment/control pairs. We *de novo* assembled these reads using Trans-ABySS to reconstruct reference transcriptomes, obtaining over 350,000 and 130,000 putative transcripts for *R*. *catesbeiana* and *X*. *laevis*, respectively. Using available genomics resources for *X*. *laevis*, we annotated over 97% of our *X*. *laevis* transcriptome contigs, demonstrating the utility and efficacy of our methodology. Leveraging this validated analysis pipeline, we also annotated the assembled *R*. *catesbeiana* transcriptome. We used the expression profiles of the annotated genes of the two species to examine the similarities and differences between the tadpole liver transcriptomes. We also compared the gene ontology terms of expressed genes to measure how the animals react to a challenge by thyroid hormone. Our study reports three main conclusions. First, *de novo* assembly of RNA-seq data is a powerful method for annotating and establishing transcriptomes of non-model organisms. Second, the liver transcriptomes of the two frog species, *R*. *catesbeiana* and *X*. *laevis*, show many common features, and the distribution of their gene ontology profiles are statistically indistinguishable. Third, although they broadly respond the same way to the presence of thyroid hormone in their environment, their receptor/signal transduction pathways display marked differences.

## Introduction

The inordinate value in having genomic information for a given species is uncontested, providing information regarding inheritance, disease, proteins, metabolites, and other regulatory molecules. However, only a miniscule fraction of eukaryotic species has more than the most basic genetic information available. For example, 8,624 genomes to date are listed in the Genomes Online Database [1] (www.genomesonline.org, Accession date: August 22, 2104) that are on-going or completed, out of an estimated 8.7±1.3 million eukaryotic species worldwide [2].

Despite concerted efforts to obtain representative genomes such as the Genome 10K project [3], sequencing the genomes of organisms of interest presents a considerable challenge in bioinformatics, and requires substantial resources that are beyond the budgets of most projects. On the other hand, high throughput sequencing of transcriptomes (RNA-seq) has the potential to rapidly and economically transform a species’ molecular knowledgebase. Transcriptomes provide information on complex biological processes, and give a picture on how the static genome behaves in dynamic environments. They enable the assessment of environmental impact factors, such as climate change—and of particular interest to our study—pollutants.

RNA-seq data are typically generated as tens to hundreds of millions of paired 75-mer to 150-mer reads. These reads are often mapped onto a completed, annotated genome scaffold for identification and quantitation [4]. In the absence of a reference genome or when the reference is poorly reconstructed or annotated, *de novo* assembly of RNA-seq reads [5–8] is an enabling technology to study the transcriptomes of non-model species [9–13]. Although the analysis of the results of a *de novo* transcriptome assembly is a nontrivial task [14, 15], coupled with a quality controlled and streamlined bioinformatics pipeline, it is a cost-effective strategy to glean biological insights. In the present work, we demonstrate the use and value of the technology to compare the liver transcriptomes of two amphibian species when exposed to thyroid hormone.

Amphibians are among the most threatened vertebrates on the planet [16]. They are also the only group where most of its members exhibit a life history that includes distinct independent aquatic larval and terrestrial juvenile/adult phases. The transition between the larval and juvenile phases requires substantial or complete remodelling of the organism (metamorphosis) in anticipation of a terrestrial lifestyle. This places amphibians in a unique position for the assessment of toxicological effects in both aquatic and terrestrial environments. Amphibians have an undeniable role as sentinel species, as a food source, and in insect control; yet over 60% of about 7,000 extant amphibian species are currently threatened or declining in numbers [16].

Despite their established importance, only one completed amphibian genome is currently available from a model diploid laboratory frog, *Xenopus (Silurana) tropicalis* [17]—a species whose natural habitat is restricted to parts of Africa [16, 17]. The most extensively used laboratory frog is *X*. *laevis*, and among amphibians, it has the largest proportion of cDNA resources available on public databases [18]. However, due to challenges in dealing with its allotetraploid nature, only recently have portions of this species’ genome become available. This species also has a natural range limited to Africa with some accidental introductions in the United States [16].

On the other hand, several relatives of the most numerous amphibian species, the “true frogs” (*Ranidae*), can be found worldwide as native or introduced species. Despite their importance as environmental sentinels and ecosystem service providers, there are only limited genomic resources for these species [19, 20].

*Xenopus* diverged from the true frogs over 200 million years ago [21]. This evolutionary divergence is accentuated by their differing life histories, behavior, and markedly different sex differentiation systems [22]. Recent evidence suggests that the innate immune system of *Xenopus* is also fundamentally different from three frog families including the *Ranidae* [20]. Furthermore, the current genomics tools developed for *Xenopus spp* are unfortunately inadequate for use with *Ranids*; *Xenopus tropicalis* reference genome representing an ineffective genome scaffold to study *Ranid* transcriptomes due incomplete or inaccurate annotation and extensive interspecies sequence divergence. A higher level of transcript annotation exists for a related species, *X*. *laevis*, due to its long-standing use as a developmental model. Despite earlier cDNA sequencing efforts [18], recent *de novo* RNA-seq experiments on *X*. *laevis* embryos discovered thousands of novel transcripts [23], further accentuating the paucity of genomic resources even for this well-studied amphibian species.

The gap in knowledge regarding postembryonic development in amphibia is even more pronounced. Previous work has established a clear dependence of thyroid hormones (THs) in amphibian metamorphosis, and although several landmark studies have characterized the genetic programs involved in select tissues (e.g. [24–28]), changes involving the liver have been less studied [27, 28].

The liver as the largest internal organ performs essential metabolic, exo- and endocrine functions, including bile production, metabolism of dietary compounds, detoxification, carbohydrate metabolism, and production of blood clotting factors and serum proteins [29]. The precise role that the liver serves in each of these biological processes changes dramatically during the TH-dependent genetic reprogramming of this organ [25, 27] from larval to juvenile form. Biochemical changes include induction of the urea cycle, albumin synthesis, and globin switching, in addition to changes in immune system function that impact the liver [30].

Here we examine the TH response of the tadpole liver tissues of two frog species, *Rana* (*Lithobates*) *catesbeiana* (American Bullfrog, *R*. *catesbeiana* henceforth) and *Xenopus laevis* (South African clawed frog, *X*. *laevis* henceforth) using RNA-seq assays. Although the species under study lack finished reference genomes, we demonstrate that *de novo* transcriptome assembly methods constitute an enabling technology for comparative transcriptomics. Reconstructing the liver transcript sequences for the two species, we annotated lists of putative protein encoding mRNA, and performed comparative analyses on gene ontology to identify common and species-specific functional processes as they relate to TH status.

## Results

We sequenced liver tissue transcriptomes of *R*. *catesbeiana* and *X*. *laevis* tadpole liver tissues exposed to the thyroid hormone, 3, 3’, 5-triiodothyronine (T3), and to the vehicle control sodium hydroxide (NaOH). Previous analysis of these transcriptomes indicated that they exhibited an appropriate response to T3, and, thus, they are representative of the TH-mediated induction process [28, 31]. We assembled transcriptomes for the two species using Trans-ABySS [8] following the protocol described in the Methods section. The putative *R*. *catesbeiana* and *X*. *laevis* transcript fragments were reconstructed in 353,253 and 134,203 contigs, respectively. These draft liver transcriptomes formed the basis of our expression level analyses and functional gene annotation. Collected sequencing data and summary statistics for transcriptome assemblies are shown in Table 1.

**Table 1:** RNA-seq data and transcriptome assembly results.

Species		# RNA-seq reads (10^6^)	Average fragment length (bp)	# Contigs	Contig N50 (bp)	Transcriptome size (Mbp)	GC content (%)
*R*. *catesbeiana*	Control / T3	347 / 472	375 / 360	353,253	1,815	429	43.3
*X*. *laevis*	Control / T3	396 / 428	394 / 376	134,203	1,596	151	41.5

Sequences were generated using 75 bp paired end reads.

We assessed the quality of the final transcriptome assemblies using 248 highly conserved core eukaryotic genes [32] (CEGs), and showed that we were able to reconstruct all CEGs for *R*. *catesbeiana*, and miss only one for *X*. *laevis*. Also, using BWA [33] and allowing for multiple alignments, we were able to align 96.1% and 94.6% of raw reads back to the assembled *R*. *catesbeiana* and *X*. *laevis* transcriptomes, respectively. These results indicate a good quality assembly, suitable for downstream biological analyses.

To examine the extent of ortholog overlap between the two species, tBLASTx was used to compare the contig sets from *R*. *catesbeiana* and *X*. *laevis*. We observed that 41% of the *R*. *catesbeiana* contigs had orthologous sequences in the assembled *X*. *laevis* liver transcriptome. Also, open reading frame analysis using TransDecoder (http://transdecoder.sf.net) indicated that 15% (51,720) of *R*. *catesbeiana* contigs had putative coding potential, similar to 14% (18,328) of *X*. *laevis* contigs.

Using BLAST tools [34], we compared our putative liver transcripts with three potential annotation resources: a collection of *R*. *catesbeiana* expressed sequence tags (ESTs), all amphibian cDNA sequences from NCBI nucleotide database [35], and *Rana clamitans* transcriptome shotgun assembly (TSA) sequences from NCBI; the non-redundant NR database of NCBI [35]; and the human protein database of Ensembl [36]. The NR database is a curated sequence database of genomes, transcripts and proteins, and aims to provide a non-redundant representation of those sequences. The amphibian cDNAs we collected from NCBI are not yet curated into NR, but most of the Ensembl human proteins are represented in NR.

The database that yielded the highest number of hits was the amphibian cDNA collection. Using those sequences, we were able to map 257,793 (73%) of *R*. *catesbeiana* and 105,030 (78%) of *X*. *laevis* contigs with BLASTn. Although BLASTx alignments to NR yielded fewer hits (129,326 for *R*. *catesbeiana* and 69,582 for *X*. *laevis*) they did provide 20,813 unique additional annotations for the *R*. *catesbeiana* contigs, and 4,607 unique additional annotations for the *X*. *laevis* contigs. Finally, BLASTx alignments to Ensembl human proteins returned 92,524 hits for *R*. *catesbeiana* and 60,243 hits for *X*. *laevis* with an expected limited number of unique hits for both species (150 and 11, respectively). Overall, using these three resources, we were able to annotate 278,799 (79%) of the *R*. *catesbeiana* and 109,649 (82%) of the *X*. *laevis* contigs in our transcriptome assemblies (Table 2).

**Table 2:** Annotation of assembled transcriptome contigs.

Contig annotations with	*R*. *catesbeiana*	*X*. *laevis*
BLASTn (amphibian)	257,793	105,030
BLASTx (NR)	129,326	69,582
BLASTx (Ensembl *H*. *sapiens*)	92,524	60,243
Total annotated combining all three methods	278,756	109,649

Three annotation approaches were taken: using BLASTn against a collection of nucleotide sequences of select amphibia, BLASTx against the non-redundant NCBI protein database, and using BLASTx against the ENSEMBL (*H*. *sapiens*) database for subsequent GO analysis.

Although the contribution of Ensembl human proteins was marginal in this scheme, alignments to this database were instrumental in functional analysis using gene ontology (GO) terms, as the collection represents the most extensively annotated data.

With the described annotation process, we were not able to observe sequence similarities between 24,554 putative *X*. *laevis* transcripts and the three resources used. We compared this remaining set with the Xenbase *X*. *laevis* 7.1 genome assembly (http://gbrowse.xenbase.org/fgb2/gbrowse/xl7_1/), and with the data reported in a recent publication evaluating *X*. *laevis* embryogenesis [23]. Of these outstanding transcripts, 21,115 matched to Xenbase genome assembly, and a further 610 had counterparts in the embryogenesis transcriptome assemblies. Thus, combining the annotation results from all resources, out of 134,203 putative *X*. *laevis* transcripts in the present study, 97.84% (131,311) matched to at least one of the relevant external resources available for this species. The remaining 2,492 putative transcripts that lack support in the literature are mostly composed of shorter sequences (N50 of 529 bp, compared to an overall *X*. *laevis* assembly N50 of 1596bp). We note that shorter sequences are relatively harder for proper alignment and annotation, and a subset of these may represent *bona fide X*. *laevis* transcripts yet to be annotated.

**Fig 1:**
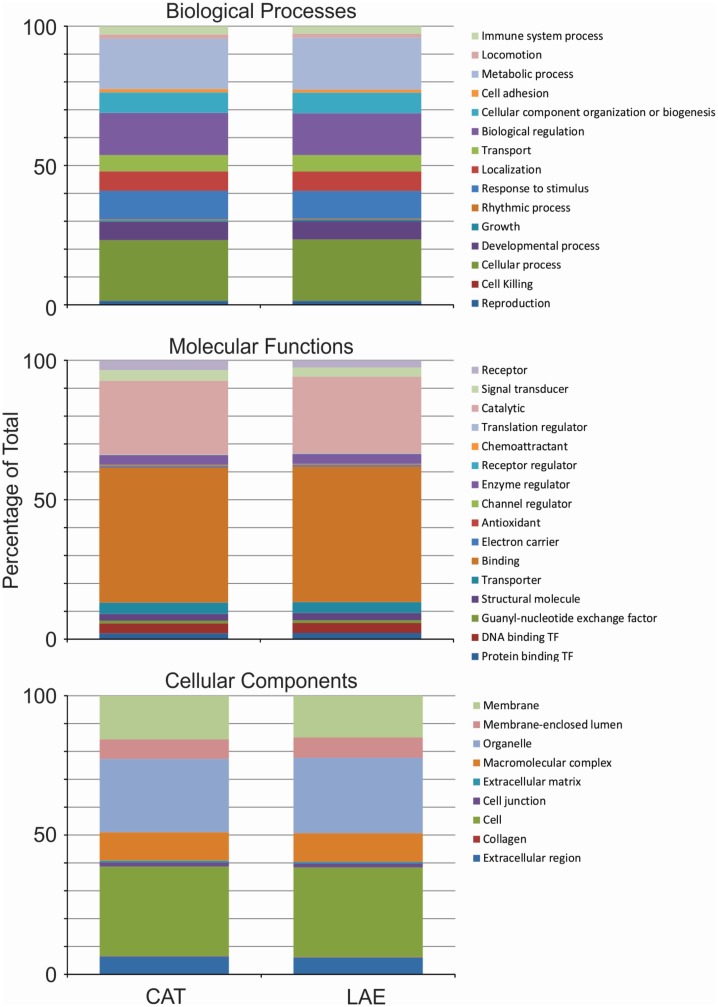
Gene ontology classification of all reconstructed *R*. *catesbeiana* and *X. laevis* liver transcripts with UniProtKB AC numbers. The two series in each stacked bar plot correspond to all *R. catesbeiana* (CAT) and *X. laevis* (LAE) transcripts.

We performed enrichment analysis of gene ontology (GO) terms on our assembled transcriptomes using the UniProtKB database (www.uniprot.org) with three perspectives: (1) Biological Processes, (2) Molecular Functions, and (3) Cell Components (Fig 1). As indicated above, for the functional annotations we used alignment of Ensembl human protein information to the putative transcripts. We subsequently mapped the Ensembl-derived human protein IDs onto UniProt entries. Using this approach, 19,514 and 17,831 unique UniProt accession identifiers (AC IDs) were generated from the 92,527 and 60,243 original Ensembl hits for *R*. *catesbeiana* and *X*. *laevis*, respectively, indicating that the relative annotations were highly similar.

We observed that the profiles comprising the Biological Processes and Cellular Components were not significantly different between the two species (Bonferroni-corrected [37] χ^2^ p-value threshold of 5%) (Fig 1 and Table A in S1 File). An example is the Immune Function category, where 1,426 and 1,174 Uniprot AC IDs were identified for *R*. *catesbeiana* and *X*. *laevis*, respectively (Table B in S2 File). Of these, 1,072 were common to both species representing 75% and 91% of the identified hits in this category, respectively (Table B in S2 File).

In contrast, initial evaluation of Molecular Functions revealed significant interspecies differences (S1 File). However, when we removed the category of receptor activity from the list, the distributions of the two species were again statistically indistinguishable. The receptor activity category revealed a higher proportion of independent UniProt AC hits in *R*. *catesbeiana* (713; Table C in S2 File) compared to *X*. *laevis* liver (471; Table C in S2 File). It is interesting to note that 410 of these hits are common to both species representing 56% and 87% of the identified hits in this category for *R*. *catesbeiana* and *X*. *laevis*, respectively (Table C in S2 File). About half of these are linked to developmental processes including angiogenesis and homeostatic processes such as protein, nucleotide, lipid, and carbohydrate metabolism. Closer inspection of those UniProt AC IDs that differ between the species indicated increased representation in *R*. *catesbeiana* of signal transduction components (246 versus 51, respectively), particularly transmembrane G protein-coupled receptors.

**Fig 2:**
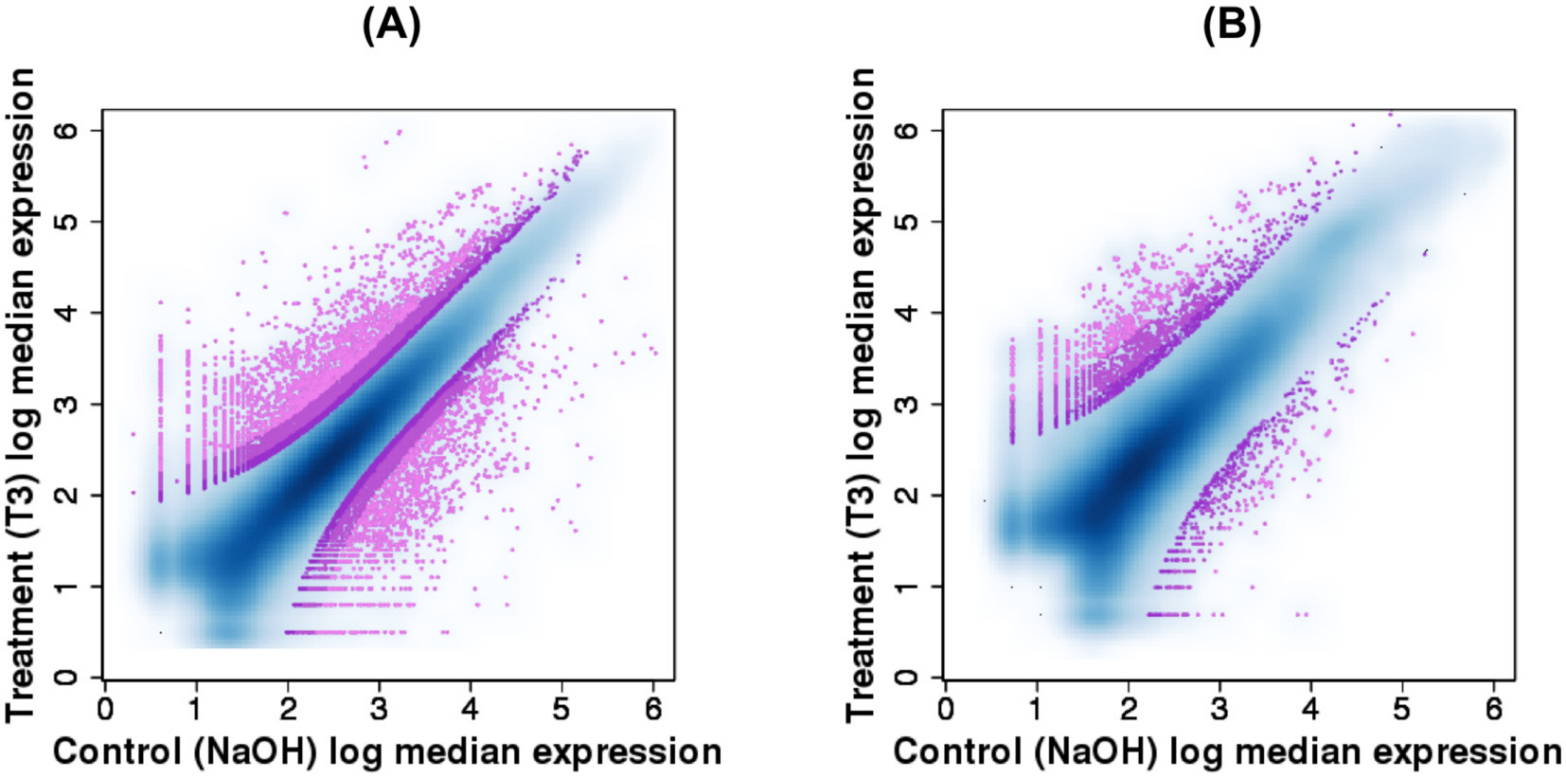
Differential expression of assembled transcripts for (A) *R. catesbeiana* and (B) *X. laevis*. Three shades of purple designate p-values of differential expression estimates 0.05, 0.02 and 0.002, lighter colours indicating lower thresholds.

Using the aligned reads and the DESeq software [38] set at three stringency levels (p = 0.2%, 2% and 5%), we analyzed differential expression between treatment-control pairs for each species (Fig 2). At a p-value threshold of 5% *R*. *catesbeiana* and *X*. *laevis* transcriptomes had 11,453 and 3,016 differentially expressed transcripts, respectively.

As an important RNA-seq validation step, seven known T3-responsive gene transcripts were evaluated in both species by qPCR (Table D in S2 File). While the RNA-seq data was derived from n = 1 per treatment, the qPCR data was derived from a minimum of 7 individuals per treatment group. There was strong concordance between the fold change values of the two independent methods supporting the integrity of the RNA-seq observations.

**Fig 3:**
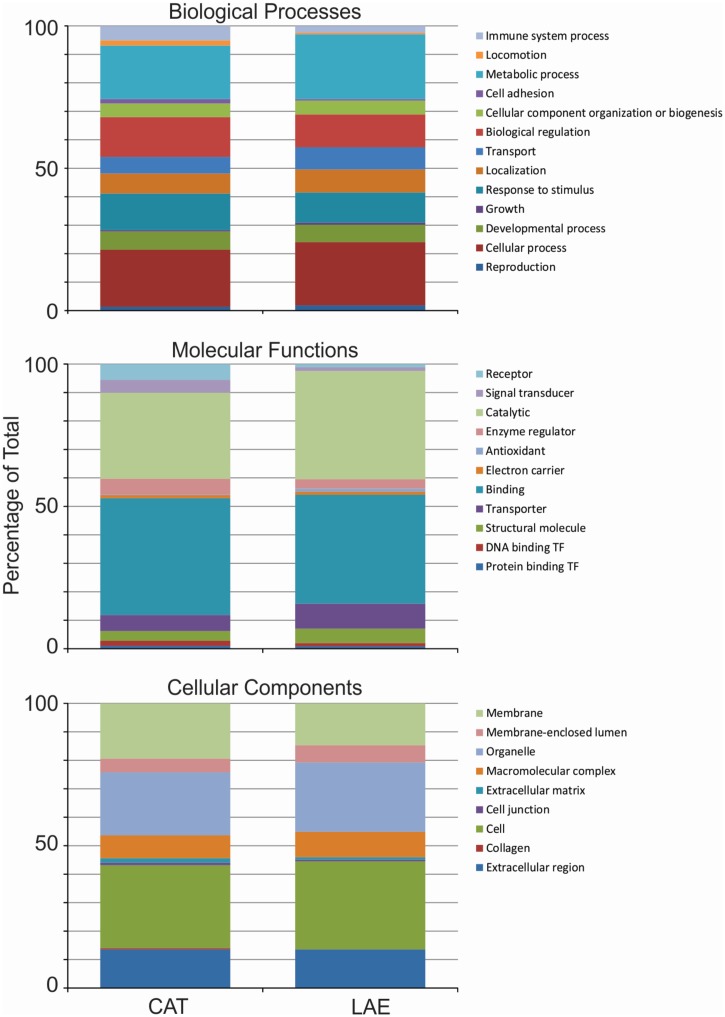
Gene ontology classification of DESeq-selected, TH-responsive *R. catesbeiana* and *X. laevis* liver transcripts with UniProtKB AC numbers. The two series in each stacked bar plot correspond to differentially expressed *R. catesbeiana* (CAT) and *X. laevis* (LAE) transcripts, with a p-value threshold of 5%.

We further analyzed the biological functions of the differentially expressed transcripts using their inferred roles in GO and pathway databases. Performing GO analysis on the differentially expressed transcripts, we observed statistically significant differences in biological processes and metabolic functions, the latter only marginally falling below the Bonferroni-corrected threshold (Fig 3, Table A in S1 File). We further performed z-tests on the relative frequencies of categories to observe that differentially expressed transcripts with functions in immune system processes differ between the two species. Of the 106 and 24 UniProt AC IDs (*R*. *catesbeiana* and *X*. *laevis*, respectively) identified by DESeq as T3-responsive, only eight were common (Table E in S2 File). This strongly contrasts with the high degree of commonality identified in the overall Uniprot AC ID profiles of the two species (Table B in S2 File). In the receptor activity category, a significant difference between species was maintained upon T3 exposure. However the nature of the profile differed with *R*. *catesbeiana* exhibiting 10 times more transcripts in this category compared to *X*. *laevis* (50 for *R*. *catesbeiana versus* five for *X*. *laevis*; Table F in S2 File). Only two sequences were common between the two species in this category (Table F in S2 File). See S1 File for detailed reports on the GO analysis and the hypothesis tests performed, and S3 File for bioinformatics details.

**Fig 4:**
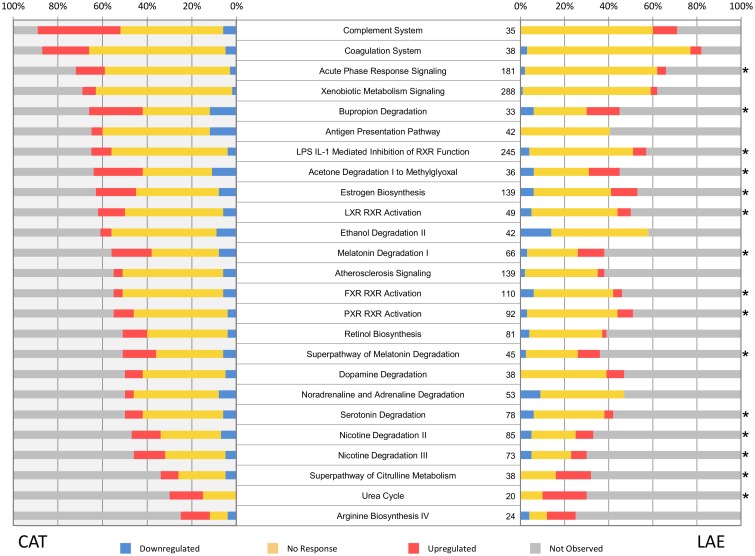
Pathway analysis for liver transcripts from *R. catesbeiana* (CAT) and *X. laevis* (LAE). Top 25 impacted pathways after TH treatment for *R. catesbeiana* ranked by the highest proportion of overall observed genes. The pathway names are indicated in the center of the figure with the total number of genes known in each IGA pathway indicated. The asterisk indicates those pathways that are found in the top 25 list of *X. laevis*. The colour coded bar plots illustrate the percentage of the total number of gene transcripts in a pathway that are downregulated (blue), non-responsive (yellow), upregulated (red) or not observed in the experiment (gray) relative to the control condition. Differentially expressed transcripts were determined using a p-value threshold of 5%.

**Fig 5:**
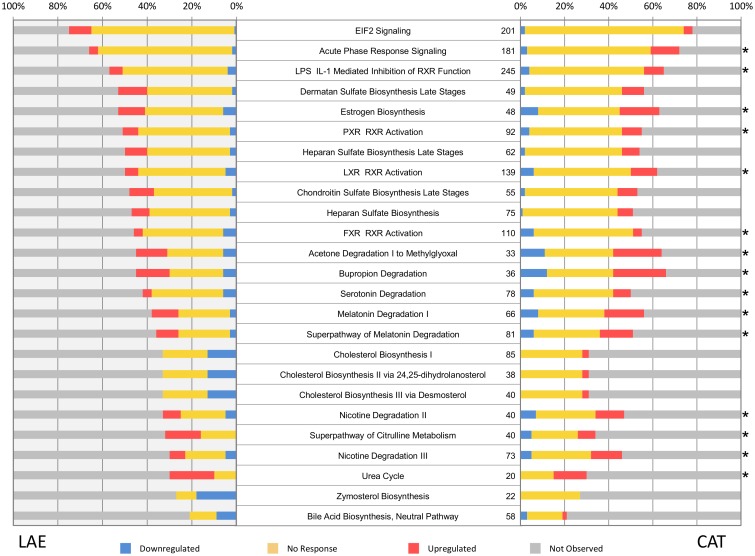
Pathway analysis for liver transcripts from *R. catesbeiana* (CAT) and *X. laevis* (LAE). Top 25 impacted pathways after TH treatment for *X. laevis* ranked by the highest proportion of overall observed genes. The asterisk indicates those pathways that are found in the top 25 list of *R. catesbeiana*. Plot details are as in the Fig 4 legend.

Further examination of the impact of T3 exposure on the liver transcriptomes was accomplished by analyzing the UniProt AC annotated transcripts of the two species with the Ingenuity Pathway Analysis (IPA) tool (Qiagen, Hilden, Germany). Figs 4 and 5 present a prioritized list of the top 25 significantly-impacted pathways of the *R*. *catesbeiana* and *X*. *laevis* liver transcriptomes organized according to the number of observed pathway components. Over half of these pathways are shared between the two species and include acute phase response signaling, several pathways involving RXR function, lipid metabolism, melatonin/seratonin degradation, urea cycle, and estrogen biosynthesis (Figs 4 and 5). Notable differences included the marked alteration of the complement and coagulation systems and antigen presentation pathways in *R*. *catesbeiana* compared to *X*. *laevis* (Fig 4) and the greater involvement of the eukaryotic initiation factor 2 (EIF2) protein translation pathway and pathways involving glycosaminoglycans and cholesterol metabolism (Fig 5). The involvement of the immune system in the T3 response of *R*. *catesbeiana* was independently supported by qPCR analysis on select immune-related gene transcripts (Fig A in S4 File).

## Discussion

Application of *de novo* sequencing and assembly approaches has enabled the direct comparison of the expressed gene information contained in two distantly related amphibian species. Both *X*. *laevis* and *R*. *catesbeiana* are pivotal species of interest in examination of the role of THs during the postembryonic metamorphic process. Although similarities in the response patterns have been implied, the extent of similarity and differences linked to their species specific natural histories have not been directly investigated. For example, *X*. *laevis* frogs remain aquatic, while *R*. *catesbeiana* frogs adopt a semi-terrestrial lifestyle around the riparian areas they inhabit. Moreover, it is known that these two species have different sex determination systems: the chicken-like ZW for *X*. *laevis*, and the XY for *R*. *catesbeiana*, similar with humans [22]. Such differences may influence their sensitivity to environmental contaminants that act through disruption of endocrine systems [28].

Our study presents a high quality assembly of the liver transcriptome of developmentally-matched premetamorphic tadpoles of both species. The quality of the transcriptomes produced is accentuated by the fact that 98% of contigs we assemble have counterparts when compared to the currently available genomic resources (www.xenbase.org) [23]. Also, the contig sizes obtained in the present study are longer than those published previously [19, 23]. Most next generation sequencing data available are currently derived from *X*. *tropicalis* due to its amenable genetics and genome resources, and the current estimated number of genes for this species is ~28,000 with an average transcript length of 1,300 bp [39]. Our observations indicated that the transcript N50 lengths for both *R*. *catesbeiana* (1,815 bp) and *X*. *laevis* (1,596 bp) species were highly consistent with this estimate. These contig sizes are approximately three-fold longer than those reported from pyrosequencing and *de novo* assembled transcriptomes of two *Ranid* species [19]. Even though the number and size of reconstructed transcripts varied between the two species, the fact that we were able to obtain close to 20,000 UniProt AC IDs suggests that the generated contig assemblies are most likely a comprehensive representation of the tadpole liver transcriptome. We think the differences in the assembly statistics are confounded by the variable size and complexity of the two transcriptomes, as well as the biology of the two species.

The results of the current study explore the limits of utility of *de novo* transcriptome assemblies for functional annotation. While the existence of the vast majority of contigs as *bona fide* transcripts is now clear, relatively few could actually be annotated sufficiently for subsequent GO analyses. Moreover, roughly 85% of the transcripts did not have putative ORFs in both species; consistent with an observation recently made by Blower *et al*. during *X*. *laevis* embryogenesis [23]. This remarkable cross-species consistency supersedes gene ploidy levels and, in fact, has been observed in investigations of human, mouse, and zebrafish where long noncoding RNAs (lncRNAs) are spliced and polyadenylated but lack protein coding ability [40–43]. Therefore more attention should be given to such ORF-negative RNAs whose implied biological significance is currently not known.

In contrast to the relative richness of genomic and transcriptomic information available for *Xenopus* species, relatively few resources are available for the *Ranidae*. The present work fills a critical knowledge gap, and provides relevant comparative information regarding the gene expression profiles of the liver in the premetamorphic tadpole with or without exposure to the metamorphosis-inducing TH. It was of great interest to establish the degree of similarity of this tissue’s responsiveness between species, and we observed that *R*. *catesbeiana* and *X*. *laevis* liver transcriptomes were highly similar to each other yielding essentially superimposable GO profiles.

The liver undergoes a substantial genetic reprogramming during TH-dependent metamorphosis, but the life histories of these two frog species suggest that the liver is likely to display marked differences in some pathways linked to their diverse ecological niches as frogs. We found that the vast majority of the transcript profiles of the livers of premetamorphic tadpoles are highly comparable to each other with the exception of the receptor/signal transduction-related pathways; particularly with respect to G protein signaling. This may translate into downstream differences in developmental outcomes, but could also contribute to differential species sensitivities to toxicants and environmental contaminants [44].

In the context of precociously-induced metamorphosis, exposure of premetamorphic tadpoles to TH results in a substantial reprogramming of the liver in both species. However, the remarkable similarity of response to hormone action extended across most pathways including lipid signaling and metabolism, the urea cycle, and pathways involving cytochrome P450 mixed function oxidases (*e*.*g*. acute phase response signaling, lipid metabolism, melatonin/seratonin degradation, and estrogen biosynthesis). All of these pathways have previously been identified as TH and/or metamorphic targets [27, 45–52].

Despite these marked similarities across the two amphibian species, species-specific differences in receptor/signal transduction pathways were maintained following TH induction. However, the most prominent species difference observed was the degree of involvement of the immune system in response to T3 exposure. Although a large number of transcripts associated with immune system functions in complement, coagulation, and antigen presentation pathways were detected in both species, there was a high degree of primarily elevated responses found in *R*. *catesbeiana* compared to *X*. *laevis*. This could represent a fundamental difference in the TH-mediated maturation of the immune system [45]. *Xenopus* species have proven to be excellent models for the comparative and developmental study of the immune system in vertebrates [53]. Unfortunately though, the functional genomics of the immune system are still poorly understood for thousands of other frog species, many of whom transition to a terrestrial lifestyle post metamorphosis, in contrast to the completely aquatic *Xenopus*. In fact, recent work on the immunomes of two frog species, *Espadarana prosoblepon* and *Lithobates yavapaiensis*, identified sigificant divergence in inflammatory response and acquired immunity transcripts relative to innate immunity and immune system development transcripts [19]. The implications of the findings presented herein and in the Savage *et al*. study [19] suggest that there are different propensities for responding to immunological cues; an important factor since many amphibian (and an increasing number of reptilian and fish) populations are severely threatened by emerging infectious diseases including *Batrachochytrium dendrobatidis* (Bd) fungus and iridoviruses [54–56]. Susceptibility to disease is known to be developmental stage dependent [45], but the factors that determine whether an organism lives or dies as a result of infection are not known. Recent work using microarrays on Bd-infected *X*. *tropicalis* [57] and iridovirus-infected fathead minnows [58] reveal that there remains much to learn about the interplay between infection and immunity, and why some animal populations are more susceptible than others to these diseases [59]. Our present work adds additional insight towards the interplay between hormone action and immune system maturation in the context of anuran development.

## Conclusions

*R*. *catesbeiana*, and *X*. *laevis* represent 260 million years of evolutionary divergence with very different genome sizes and ploidy levels. The present study demonstrates that the *de novo* transcriptome sequencing and assembly method we report is a successful approach that provides a valuable foundation from which to tackle critical biological issues that can extend beyond what can be gleaned from a restricted number of laboratory-reared species available, as well as support investigations related to comparative biology. As such, our methodology can act as a guideline for studying species that currently lack an associated reference genome.

## Materials and Methods

### Ethics approval

Premetamorphic *R*. *catesbeiana* tadpoles were locally caught (Victoria, BC, Canada) under a BC Ministry of Forests, Lands and Natural Resource Operations permit VI11-71459 while X. laevis tadpoles were raised at the University of Victoria Aquatics Facility. Animal husbandry was carried out in accordance with the guidelines of the Canadian Council on Animal Care. The University of Victoria animal care committee specifically approved this study.

### Sample collection

Premetamorphic *X*. *laevis* (NF stage 54) and *R*. *catesbeiana* (TK stage VI-X) tadpoles were exposed to 400 μM NaOH (CAS 1310-73-2, ACP Chemicals Inc., Montreal, QC, Canada) or to 10 nM 3,5,3′-triiodo-L-thyronine (T_3_; CAS 6106-07-6, Sigma-Aldrich Canada Ltd., Oakville, ON) in NaOH vehicle for 48 h as described previously (Veldhoen et al., 2014). Treated tadpoles of both species were euthanized using 0.1% (w/v) tricaine methanesulfonate in dechlorinated municipal water containing 25 mM sodium bicarbonate. Liver tissue was isolated and preserved in RNA*later* following the manufacturer’s protocol (Life Technologies Inc., Burlington, ON) and hepatic total RNA extracted using TRIzol reagent as described previously [31]. RNA-seq libraries were generated from 8 to 11μg of total RNA with RIN scores in the range from 6.6 to 7.2, using superscript random primer for double strand cDNA synthesis (SPCL Kit, Invitrogen, Waltham, MA). Generated libraries were sequenced using the HiSeq 2000 paired-end sequencing platform as per the manufacturer’s instructions (Illumina Inc., San Diego, CA) to generate 2x75 base pair reads.

### Transcriptome assembly

Each of the four libraries was assembled separately with Trans-ABySS [8] over a range of k-mer sizes (every other k between 38 and 74 bp). Trans-ABySS was selected for its robust and competitive performance in assembling transcriptomes (see [60] for a recent comparison to other available transcriptome assemblers). The final assemblies of the two conditions (T3 and NaOH) were concatenated separately for each species. In these assemblies redundant sequences were removed by aligning contigs to themselves (with BWA [33], default parameters); allowing no alignment gaps. Shorter contigs were tagged for removal when they had at least 95% match to a longer contig. Further, SGA [61] was used on the final set of contigs to improve the contiguity and remove remaining redundant sequences by performing overlap-based contig extensions.

We used BioBloom Tools [62] to screen for possible bacterial and viral contaminations in the original reads, and filtered out contigs associated with these reads. The remaining contigs were used as the final set of assemblies for annotation and differential expression analysis. Assembly statistics in Table 1 refer to these final assemblies.

Tool versions, parameters and command lines for transcriptome assembly are detailed in the S3 File. The file includes scripts to reproduce our analysis.

### Open reading frame analysis

We used TransDecoder (http://transdecoder.sf.net) with the default parameters to predict the open reading frames in the final assemblies for both species.

### Comparison of transcriptome assembly contigs with existing *X*. *laevis* assemblies

The *sensitive* option of Bowtie [63] was used to map the putative *X*. *laevis* transcripts to the Xenbase *X*. *laevis* 7.1 genome assembly and the transcriptome assemblies from a study on *X*. *laevis* embryogenesis [23]. We required at least half of the entire length of our *X*. *laevis* transcriptome contigs to be used in the alignments.

### Differential expression analysis

Bowtie [63] with *multiple alignment* option, was used to separately align the control and treatment raw reads to the final assemblies in each species. To estimate the relative expression levels of reconstructed transcripts at gene level, we generated the raw median fold-coverage for all contigs based on these read alignments. Using DESeq [38] in *blind* mode and with *fit-only* option, we selected the final set of differentially expressed genes with p-value threshold 5%.

### Transcript annotation

Contigs were aligned using the BLASTx or BLASTn programs from the BLAST+ software package [64] against three reference databases: (1) the NCBI non-redundant (NR) database (retrieved 24 March 2014); (2) a collection of all amphibian transcript sequences, including mRNA from *X*. *tropicalis* and *X*. *laevis*, as well as *R*. *catesbeiana* expressed sequence tags (EST) and TSA data from the green frog, *R*. *clamitans*, in GenBank (retrieved 25 February 2014); and (3) the Ensembl *homo sapiens* cDNA database from the GRCh38 genome assembly (retrieved 9 June 2014). To identify sequence orthologs between the species, tBLASTx was used with the *R*. *catesbeiana* contigs to query the *X*. *laevis* contigs generated from the livers in the present study. Alignments with a minimum E-value of 1x10^-5^ were considered, and the top alignment from each of the three databases was retained and merged. Preference was given to the Ensembl result for identification purposes due to the extensiveness of its associated annotations, while the result from the set of amphibian transcript sequences provided independent confirmation of the veracity of *de novo* assembled sequences.

### Gene ontology analysis

Gene ontology (GO) analyses were performed using the Ensembl-annotated data. The Ensembl IDs were mapped to the corresponding UniProt IDs using ID mapper (www.uniprot.org). The unique UniProt IDs were used as input to retrieve the GO data for molecular function, cellular component, and biological process categories and subcategories within. In performing the gene ontology analysis, we used the UniProt database release 2014_07, dated 9 July 2014.

The normalized stacked bar plots in Figs 1 and 3 show the relative frequencies of categories over three domains of classification: (1) Biological processes describe molecular activities within cells, tissues and organs (15 categories); (2) Molecular functions relate to chemical events, such as catalysis or enzyme activities (21 categories); and (3) Cellular components refer to where in a cell or its extracellular environment a particular transcript is active (nine categories). In plotting the figures, for molecular functions, we filtered out categories that had less than five representative transcripts for either species. The other two domains had all their categories meeting this criterion. In performing statistical significance tests, we considered all categories in each domain.

To establish statistical similarities between the relative frequencies of categories in both species, considering all or differentially expressed transcripts, we performed a series of χ^2^ tests. We applied a Bonferroni correction [37] factor of 6, for three domains of classification and two sets of comparisons (for all and for differentially expressed transcripts). We applied the same coefficient when we excluded the receptor activity from the list of categories in molecular functions. We performed statistical similarity tests for the relative frequencies of 45 categories for two sets of comparisons. However, seven of them had zero occurrence in the differentially expressed list of transcripts, hence were not tested for significance. To reflect the number of tests we performed, we used a Bonferroni correction factor of 83.

Frequencies of observation of each category in all three domains, and the results of our hypothesis tests are provided in the Supplementary Materials (S1 File).

### Pathway analysis

Biological pathways were generated using the Ingenuity Pathway Analysis (IPA, QIAGEN Redwood City, www.qiagen.com/ingenuity). Uniprot IDs of differentially expressed genes and their associated log fold change ratios are used as the input for IPA. In the case of redundancies, which occurred when multiple contigs are annotated with the same ID, log fold change of the longest contig is considered representative for that gene. For each pathway, a p-value is calculated using the Fisher’s exact test against the null hypothesis that genes from that pathway are appearing in our list randomly. These p- values are then adjusted using the Benjamini-Hochberg correction. The analysis was performed with the IPA 2014 Spring release.

### Quantitative real-time polymerase (qPCR) validation

Independent validation was performed on vehicle and T3-exposed tadpoles (n = 7–16) using qPCR as described previously in Veldhoen et al., 2014. All primers were subjected to a rigorous three tier quality control (QC) procedure to ensure accurate detection and optimum performance (Veldhoen et al., 2014). The new immune system components primers and qPCR assay conditions are referred to in Table G in S2 File. Statistical significance was determined using the Mann-Whitney U test using R Studio software (www.R-project.org).

## Supporting Information

S1 File An Excel spreadsheet reporting the results of the gene ontology (GO) analysis. First three sheets show category counts for the biological processes, molecular functions, and cellular components. The last sheet shows Table A, reporting the results of statistical tests performed to compare overall distributions, and the relative frequency of each category.
(XLSX)

S2 File Tables B—G. (XLSX)

S3 File Further details on the transcriptome assembly process, custom scripts and versions of the software used. (TAR)

S4 File Fig A. (PDF)
