# Supplementary material for: De novo Transcriptome Assemblies of Rana (Lithobates) catesbeiana and Xenopus laevis Tadpole Livers for Comparative Genomics without Reference Genomes
Source: PLoS One. 2015 Jun 29;10(6):e0130720. doi: 10.1371/journal.pone.0130720 (PMC4488148; doi:10.1371/journal.pone.0130720)
Supplement: S4 File — (PDF) [file pone.0130720.s004.pdf]

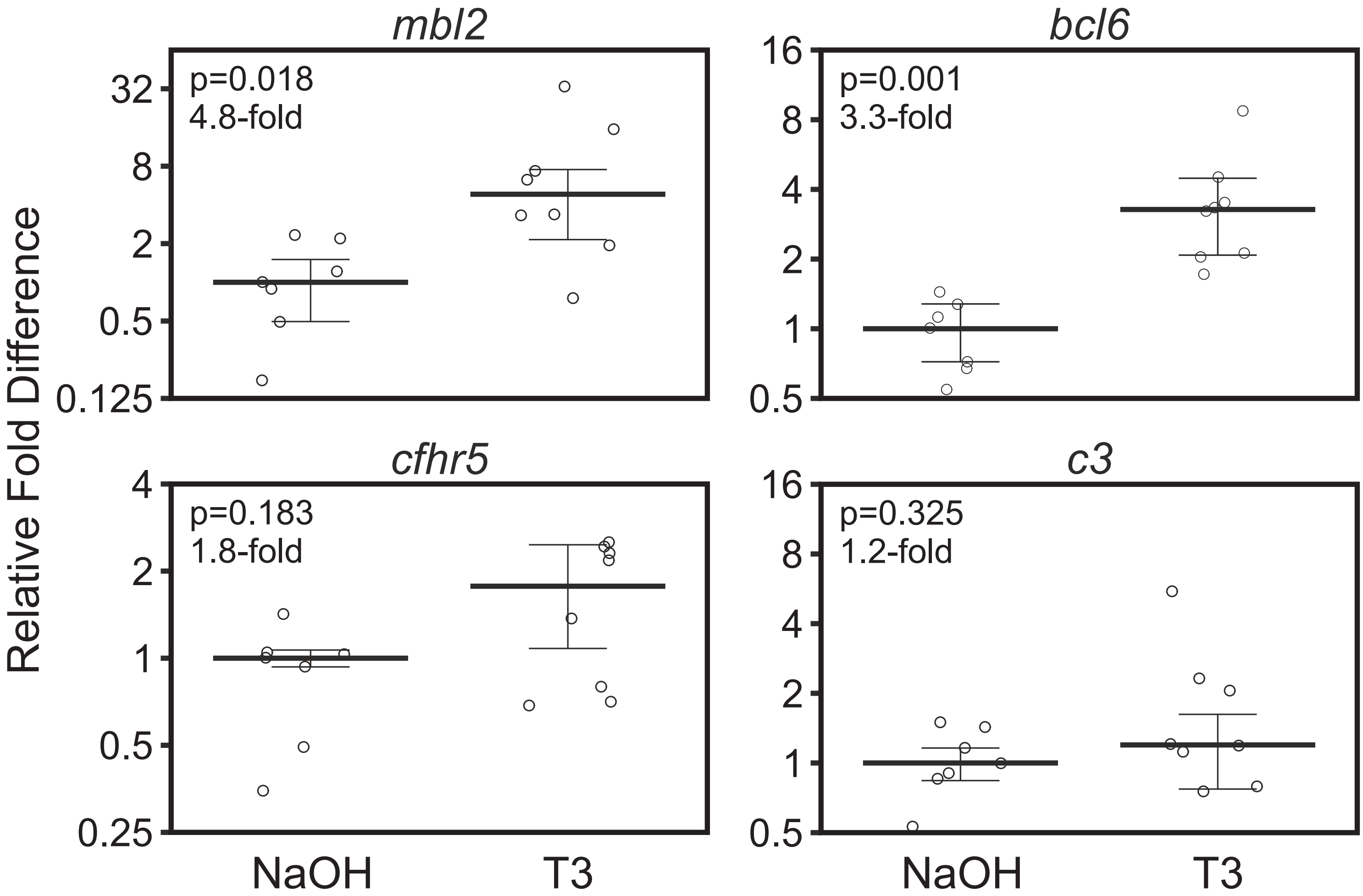

Supplemental Figure 1. qPCR analysis of immune system components in *R. catesbeiana* tadpoles treated with vehicle control (NaOH) or hormone (T3). Individual animals are depicted by the open circles with the median values (black horizontal bar) and median absolute deviation (whisker) shown.
